# Puerperium experience and lifestyle in women with gestational diabetes mellitus and overweight/obesity in China: A qualitative study

**DOI:** 10.3389/fpsyg.2023.1043319

**Published:** 2023-03-15

**Authors:** Jing He, Kaili Hu, Cui Xing, Binghua Wang, Tieying Zeng, Hui Wang

**Affiliations:** ^1^Department of Nursing, Tongji Hospital of Tongji Medical College of Huazhong University of Science and Technology, Wuhan, China; ^2^School of Nursing, Tongji Medical College of Huazhong University of Science and Technology, Wuhan, China

**Keywords:** gestational diabetes mellitus, overweight, obesity, puerperium, lifestyle, qualitative research

## Abstract

**Introduction:**

Women with overweight or obesity and gestational diabetes mellitus (GDM) are at a high risk of developing type 2 diabetes mellitus (T2DM) and other metabolic diseases. Healthy postpartum lifestyles in women with GDM are important for effectively preventing early T2DM occurrence; however, few studies and guidelines focus in China on this issue.

**Aims:**

This qualitative study aimed to understand the puerperium experience and lifestyle of women with overweight/obesity and GDM.

**Methods:**

A face-to-face, in-depth, and semi-structured interview was conducted using a hermeneutical phenomenology method to collect data that were analyzed through thematic analysis.

**Results:**

Out of 61 recruited women with overweight/obesity and history of GDM, 14 women underwent an interview and provided detailed descriptions of their lifestyle experiences during puerperium. The interview data were used to generate four themes—puerperium dietary behavior, weight perception and “confinement” behavior, family support, disease knowledge, and perceived risk—and nine sub-themes.

**Conclusion:**

Unhealthy lifestyles, misconceptions about food, the conflict between physical activity and confinement behavior, a lack of social and family support, and low awareness of disease risk are all common among overweight/obese women with a history of GDM. Thus, we emphasized that healthcare providers should provide continuous preventive care from pregnancy to postpartum and promote long-term health in high-risk populations with a history of GDM associated with overweight/obesity.

## Introduction

The prevalence of gestational diabetes mellitus (GDM) continues to rise worldwide due to lifestyle changes, increasing women’s body mass index (BMI), advanced age, and other factors (Carracher et al., 2018). GDM was defined as hyperglycemia first diagnosed during pregnancy, with a blood glucose level lower than that of gestational significant diabetes mellitus (Metzger et al., 2010). According to the International Association of Diabetes in Pregnancy Study Groups (IADPSG) diagnostic criteria, which are routinely monitored at gestational 24–28 weeks, the prevalence of GDM in China from 2012 to 2016 was 17% (14–20%) (Narenqimuge et al., 2018; Chinese Medical Association Department of Obstetrics and Gynecology et al., 2022). Risk factors for GDM include non-modifiable factors such as parity, family history of diabetes, and polycystic ovary syndrome (PCOS), as well as modifiable risk factors such as being overweight/obese, dietary habits, physical activity, and psychological factors (McIntyre et al., 2019).

In recent years, the proportion of overweight and obese women of childbearing age has increased significantly due to their unhealthy eating habits and lack of physical activity. The risk of developing GDM is more than twice as high among overweight and obese pregnant women than that among non-obese women (Wang et al., 2017). Furthermore, the combination of overweight/obesity with GDM may exacerbate these adverse outcomes caused by GDM alone (Wu et al., 2022). Women with low levels of physical activity were more than four times more likely to develop GDM than those with high physical activity levels, classified into areas of life as sedentary, mild, moderate, and vigorous (Gascoigne et al., 2023). Moreover, there was a marginally significant association between unhealthy eating patterns, depressive symptoms in early pregnancy, and the risk of GDM (Minschart et al., 2021; Haghighatdoost et al., 2023). Therefore, reasonable weight loss, a healthy diet, and regular exercise are modifiable risk factors that can help prevent GDM, which has both short-term (e.g., preeclampsia and macrosomia) and long-term effects (e.g., diabetes and premature cardiovascular disease) on women and their offspring (Metzger et al., 2008; Zhu and Zhang, 2016; Zhou et al., 2020).

Metabolic abnormalities in GDM are associated with increased insulin resistance and β-islet cell dysfunction in pregnant women (Batchuluun et al., 2018). Meanwhile, the study demonstrated a strong relationship between measures of insulin sensitivity, including fasting glucose and insulin, and body weight (Gao et al., 2022). Furthermore, postnatal weight gain has been identified as an important risk factor for obesity and the development of type 2 diabetes mellitus (T2DM) (Friedman et al., 2008). As reported in the observational study, no significant improvement in insulin sensitivity, body weight, or body composition was noted in women with GDM within one year postpartum (Friedman et al., 2008). These findings suggest the importance of postnatal lifestyle interventions for women with GDM. The Diabetes Prevention Program (DPP) has shown that postpartum lifestyle changes can result in weight loss, particularly in the first year postpartum, and delay or prevent the onset of type 2 diabetes (Ely et al., 2017). Therefore, a healthy postpartum lifestyle and weight recovery in overweight/obese women with GDM are important for preventing or reducing the occurrence of metabolic diseases.

Besides, maternal insulin sensitivity increases rapidly and dramatically within days after delivery, when insulin requirements are roughly half what they were before delivery (ElSayed et al., 2023a). The islet cells need to make new assessments and adjustments to meet the body’s insulin requirement (ElSayed et al., 2023a). Puerperium is the “window phase” of recovery of glucose metabolism in women with GDM, particularly those who were overweight/obese before pregnancy (ElSayed et al., 2023b). Thus, the early postnatal period for initiating and sustaining lifestyle changes is the “window period” for recovery of glucose metabolism and islet function in women with GDM, particularly those who were overweight or obese prior to pregnancy. The postpartum period is also crucial for healthcare professionals to offer advice and support to women with GDM, considering that changes in lifestyle (such as a healthy diet and increased physical activity) have been shown to prevent the further development of T2DM (Aroda et al., 2015; ElSayed et al., 2023b).

However, studies have found it difficult for women with GDM to adopt a healthy lifestyle after delivery (Krompa et al., 2020; Sharma et al., 2021). Teh et al. (2021) studied women with GDM in Singapore and found that most participants had a low awareness of future diabetes risk, unhealthy postnatal diets, and insufficient physical activity. The lack of culturally appropriate health education and social support may impact the healthy postpartum lifestyle of women with GDM (Neven et al., 2022). Therefore, understanding the postnatal lifestyle of overweight/obese women with GDM and identifying unhealthy behaviors will help lay the foundation for targeted care measures for these high-risk women.

GDM-related studies and clinical treatment in China have been focused on prenatal screening and prenatal care, while postpartum screening and sustained postnatal lifestyle care and intervention have been lacking. Furthermore, the postpartum lifestyle of Chinese women is also influenced by the cultural characteristics of confinement behavior. Therefore, the purpose of this study was to explore puerperium experiences and lifestyles of women with recent GDM and overweight/obesity. Furthermore, their thoughts were also analyzed based on postpartum blood glucose testing, weight changes, and expectations for postpartum help. The research results can provide a basis for developing culturally sensitive medical services and interventions for overweight/obese women with GDM during their puerperal period in China to prevent chronic diseases, particularly for younger T2DM.

## Materials and methods

### Research design

The study followed the consolidated criteria for reporting qualitative research (COREQ), a 32-item checklist for interviews and focus groups (Tong et al., 2007). Under the practices of “confinement” in traditional Chinese culture (Heidegger’s, 2010), hermeneutical phenomenology method was used to explore and understand the lifestyle and experiences of overweight/obese women with GDM in puerperium. The true meaning of existence and the underlying structure of being are presented through interpretation to understand existence and living in itself. We focused on comprehending how each individual’s unique experience is a way of existing. Face-to-face semi-structured interviews were conducted to understand participants’ lifestyles, dietary behavior, physical activity, adjustment process, disease risk perception, and expected help.

### Setting and participants

Women were recruited when they returned to the hospital for postnatal examination through follow-up of parturitions in the Department of Perinatal Medicine, Tongji Hospital, affiliated with Tongji Medical College, Huazhong University of Science and Technology, China. The study inclusion criteria are as follows: overweight/obese (overweight considers a BMI of 24–27.9 kg/m^2^, whereas obese considers a BMI of ≥28 kg/m^2^) before pregnancy, diagnosed with GDM, and re-visited the hospital for postpartum examination within 6–8 weeks of puerperium. The interview took place in an office at the obstetrics clinic, in a quiet environment, with only two people: the participant and the researcher. Before the interview, the researcher assisted the participants in completing a postnatal examination and establishing familiarity.

### Data collection

Face-to-face semi-structured in-depth interviews were conducted once to collect data, and audio recordings and field notes were used to document the data between April and June 21, 2022. The formal interviews lasted approximately 60 min, averaging 52.6 min. Data saturation occurs when no new information appears in the content acquired during the interviews. In this study, data saturation was noted during the interviews of the 13th–14th participants, and then the interview ended. Two researchers kept the data separately (JH and CX). In order to protect the participants’ privacy, all participants were assigned a subject ID (e.g., C1 = Participant 1). The women with GDM were requested to describe their experiences and true feelings about their lifestyle during the puerperium, as well as their perceptions of weight and disease risk.

One researcher, a doctoral candidate in nursing who focused on qualitative research and a certified psychotherapist, deeply communicated with participants to collect information. Another researcher with 16 years of nursing experience worked as the head nurse at an obstetric clinic. During the interviews, researchers used various techniques, such as empathy, repetition, clarification, induction, and summary, during the interviews to obtain in-depth information.

### Ethical consideration

This study was approved by the Medical Ethics Committee of Tongji Hospital, Tongji Medical College of Huazhong University of Science and Technology (ID: TJ-IRB20220445). Before the interviews, the researcher introduced the research content and obtained signed informed consent from all participants.

### Data analysis and interpretation

Two researchers (JH and CX) collated, analyzed, and summarized the recordings and field notes within 24 h of the interview. The recordings were transcribed using computer software (Lenovo Voice Assistant, Lenovo LTD, Beijing, China). The two researchers listened to the recordings repeatedly, making supplementary notes on details, such as modals, pauses, and emotions. The transcribed texts were uploaded to NVivo 12 (QSR International, Doncaster, Australia) to store, manage, and analyze the data.

Thematic analysis was used for data analysis, and the philosophical paradigm was constructivism (Braun and Clarke, 2006). Inductive analysis was used in the data encoding process and the description of the process of interpretation. In the description, the data were simply organized to show patterns in semantic content and to summarize. Attempts were made during interpretation to theorize the meaning of the patterns as well as their wider meanings and implications. Thematic analysis is divided into six phases: familiarizing oneself with the data, generating initial codes, searching for themes, reviewing themes, defining and naming themes, and producing the report (Braun and Clarke, 2006).

## Results

Table 1 depicts the demographic characteristics of the 14 participants. The average age was 29.93 years. Of the 14 participants, 13 were primiparas, and 1 was multipara. The mean BMI before pregnancy was 26.12 kg/m^2^, the mean gestational weight gain was 17.36 kg, and four had a family history of diabetes. Additionally, 12 and two of the 14 women were overweight and obese before pregnancy, respectively. Besides, excessive gestational weight gain was observed in 12 women.

**Table 1:** Demographic information of the participants.

ID	Age,years	Gravid	Parity	Gestational weeks + days	Pre-BMI	GWG, Kg	Family history of diabetes	Education level
C01	30	2	1	39 + 5	24.34	20	Yes	College
C02	37	2	1	36 + 6	25.64	15	No	Secondary school
C03	25	1	1	39	27.89	8.5	No	Bachelor
C04	27	2	1	39	31.11	15	No	College
C05	37	3	2	36	25.78	11.5	No	College
C06	34	1	1	40 + 2	29.30	15	No	Bachelor
C07	28	1	1	39 + 1	24.22	23	No	Bachelor
C08	27	1	1	40 + 3	27.18	16	Yes	College
C09	30	3	1	37 + 6	24.12	18	No	Bachelor
C10	26	2	1	39	24.09	22	Yes	College
C11	29	2	1	37 + 6	26.03	22	Yes	Bachelor
C12	30	1	1	40	24.97	20	No	Bachelor
C13	26	1	1	38 + 2	26.14	18	No	College
C14	33	1	1	40	24.91	19	No	Bachelor

Pre-BMI, Pre-pregnancy BMI; GWG, gestational weight gain.

After analysis and coding of the interview text, 1,173 nodes were identified. By using these nodes, four primary themes (puerperium dietary behavior and feelings, weight perception and “confinement” behavior, family support, and disease knowledge and perceived risk) and nine sub-themes were generated. Table 2 lists the themes, sub-themes, and corresponding quotes.

**Table 2:** The overview of themes, sub-themes, and quotes of women with recent GDM and overweight/obesity during puerperal experience and lifestyle.

Theme subtheme	Selected relevant quotations
Theme 1: Puerperium dietary behavior	
1.1. Postpartum dietary changes	*“I stopped controlling my weight and diet. Slowly, I will do something as now I am waiting for vital energy and blood to replenish. Do not worry.” (C09)*
	*“They (mother-in-law and maternity matron) were always pushing me to eat all kinds of foods.” (C05)*
	*“I have extra meals between meals. I am afraid of eating too much. However, I am concerned that my baby’s nutrition is not enough, so I need to eat a lot.” (C01)*
	*“I am truly tired as I am taking care of my baby all day long, and lack of sleep.” (C11)*
1.2. Diet culture	*“Peanuts, medlar, red beans, and brown sugar, boiled together, which enrich the blood (according to folklore, eating red food can nourish the blood).” (C12)*
	*“When I went home, my mother-in-law made this soup daily, and I drank a lot (approved). Besides, there is a lot of meat, which I feel I ate slightly more than I did during pregnancy (happy smile).” (C03)*
	*“I do breastfeeding, and I feel so hungry that I eat a lot of rice.” (C10)*
	*“The baby’s jaundice could be aggravated by my becoming inflamed (through breast milk). I feel a strange sensation in my throat and a slight cough, and I am afraid to drink milk (which increases inflammation). Many taboos (do not eat these things). For example, ginger and garlic.” (C02)*
	*“Hot fruits will be steamed for extra meals. I cannot eat cold, but when out of ‘confinement’ (approximately 30 days), I can eat at room temperature fruits.” (C06)*
Theme 2: Weight perception and “confinement” behavior	
2.1. Weight awareness	*“I am feeding (for baby) every day. My mother hopes me to eat more. My goal is still to be thin after the baby is weaned.” (C02)*
	*“I could not fit into my old dresses and lost interest in new ones. Now it is embarrassing for me to look at everything. I look ugly.” (C12)*
	*“I do not want to eat anything except three meals. Sometimes I am hungry, my stomach hurts, and I may drink milk. I feel shame about my weight. (C14)”*
	*“I was fat before I got pregnant. Pregnancy itself means gaining weight. But I feel healthy now, and slowly I will lose weight later.” (C01)*
2.2. Physical activity and “confinement” behavior	*“My mother asked me to lie in bed for long periods, flat on my back, and not move. She also did not allow me to play with my phone while lying down. She said lying down helps the uterus return to its original position, which I cannot accept.” (C13)*
	*“Now I dare not exercise too much for fear of affecting the wound.” (C09)*
	*“I was active during pregnancy to control my blood glucose, but I do not need that anymore.” (C10)*
	*“I only feed the baby; otherwise, it is the matron who takes care of the baby. I felt so tired after delivery that I lied down and rested every day. I wanted to do some activity, but I am tired.” (C08)*
Theme 3: Family support	
3.1. Partner support	*“I said that I was overweight. My husband is also quite funny as he said that he will wait for me to lose weight together after ‘confinement.’ He makes me laugh.” (C13)*
	*“My husband would make me eat a variety of healthy food. However, if he is at work, I can only order outside food.” (C08)*
3.2. Family influence	*“My mother-in-law always said that the baby did not eat enough as my milk is not enough. I was asked to eat a lot of food. We always have our differences, and I often cry.” (C01)*
	*“Mother-in-law asked me to eat more food including more meat. Otherwise, I do not produce milk, and the baby needs breastfeeding.” (C07)*
Theme 4: Disease knowledge and perceived risk	
4.1. Risk perception	*“I worry about my future diabetes, but I do not think I can control it. My mother-in-law does not care about anything. She cooks something you do not like and does not know what food will raise your blood sugar quickly.” (C06)*
	*“The doctor always told me I was overweight. I am not going to get diabetes as I know how terrible it is” (C04)*
4.2. Emotion of checking OGTT	*“I do not like that sugar water, and I feel vomiting while drinking.” (C05)*
	*“It is scary to draw blood samples four times a day as it hurts.” (C06)*
	*“I did not even think to test (OGTT) again. However, the doctor gave me this test. Lactation is not as tightly controlled (blood glucose) as pregnancy. If you starve yourself, your breast milk will suffer, and it is not a price worth paying.” (C08)*
4.3. Knowledge acquisition	*“I do not know anything about postpartum diet management as no medical staff told me about it. Now my purpose is how to feed the baby better, how to produce breast milk, and how do I eat.” (C09)*
	*“When I was discharged, no medical staff told me that I needed to continue monitoring my blood glucose levels and retest after delivery.” (C12)*

### Theme 1: Puerperium dietary behavior

*Postpartum dietary changes* and cultural background affect the recovery of blood glucose in women with GDM who are overweight/obese and face significant challenges (1.1). Most overweight/obese women with GDM were unaware of the need for diet management after delivery; they often ate more than they did during pregnancy, which is also related to cultural customs during the puerperal period. Furthermore, the public believes there is a lack of vital energy and blood during postpartum; thus, more nutritious foods are needed to repair the body. Other women increased their food intake because they believed their breast milk would provide more nutrients to their babies. Puerperium is a period when mothers learn and adapt to caring for their babies. Women with GDM have limited time for lifestyle changes because they constantly care for their babies, including breastfeeding.

There is a difference between the *diet culture* in Chinese confinement customs and the healthy dietary needs to prevent diabetes (1.2). Drinking soup is widely believed to promote breast milk production apart from postpartum recovery. Soup is mainly a high-fat and high-sugar diet, rapidly increasing blood glucose and negatively affecting the function of islet cells. Additionally, this may cause a lack of fresh vegetable intake. The participants had a unique understanding of the impact of food on breast milk, and their maternal diet was constrained by traditional thinking. Most women said they should not eat cold food, especially fruits, during confinement. Other women increase their food intake because they believe that their immunity will be weakened if they do not eat enough during the puerperal period. These culture-related dietary perceptions and habits are not conducive to postpartum weight and blood glucose recovery.

### Theme 2: Weight perception and “confinement” behavior

All participants described their *weight awareness* and shared their struggles and conflicts with maintaining postpartum weight on the interview day (2.1). Some people avoided answering questions about weight and emphasized that they should not lose weight to ensure adequate nutrition for their babies during breastfeeding. Some women reported weight shock and body image anxiety. Most participants expected to improve their body image after delivery, but no action was taken to change their weight. Further, they shared their feelings of pressure and frustration in recovering their pre-pregnancy weight. Other overweight women thought they were healthy and accepted their current weight status because it is normal to gain weight during pregnancy.

All participants showed similar inactivity behaviors after delivery, and *physical activity and “confinement” behavior* were linked together (2.2). Physical activity is a healthy behavior that plays an important role in preventing T2DM. However, the need for physical activity in women with GDM and overweight/obesity is poorly understood. They believe that postpartum is more about resting and avoiding physical activity to restore strength and prevent future diseases. During “confinement,” the patient is urged to only stay at home, maintaining a prostrate position for a long time, which is considered conducive to uterine rejuvenation. For some women, the importance of physical activity is thought to be related only to blood glucose control and is no longer needed postpartum. Other people hired matrons to help with household chores and childcare at home or in maternity centers. There was a lack of routine walking during puerperium. A gap was observed between willingness to increase physical activity and its implementation.

### Theme 3: Family support

The care provided by spouses and other family members was also crucial. *Partner support* is the most important source of support for women with GDM and overweight/obesity in the puerperium, considering reasonable psychological support (3.1). Women with GDM and overweight/obesity reported that their partner assisted them in providing a healthy diet and psychological support after delivery, which can reduce postpartum negative emotions and put their health in the first place. Simultaneously, the attention and companionship of spouses can promote their physical activity.

In Chinese culture, most women in “confinement” are assisted by their mothers-in-law to care for the mothers and babies. Mothers-in-law play an important role in *family influence*, and they use their traditional ideas in modern maternal life (3.2). The traditional dietary attitudes of mothers-in-law have more negative impacts on the health of women with GDM and overweight/obesity.

### Theme 4: Disease knowledge and perceived risk

The study found that women with GDM and overweight/obesity interviewed after delivery had a lack of *risk perception* to prevent future diabetes (4.1). We found that in traditional Chinese beliefs, pregnancy requires more nutritious food and fruits, leading to excessive weight gain during pregnancy. Furthermore, most overweight/obese women with GDM did not undergo glucose management or monitoring during pregnancy. Perceptions during pregnancy pose challenges for changes in postpartum lifestyles. Some participants expressed concern about the effect of blood glucose on the fetus during pregnancy, believing that GDM is a pregnancy-induced change that returns to normal after delivery. Only a few women knew that a history of GDM puts them at a higher risk of developing diabetes later in life. However, they were unaware of the impact of a postpartum lifestyle and keeping gestational overweight/obese. Although they intend to maintain a healthy lifestyle, most women do not do so or find it difficult to make changes.

Women with GDM and overweight/obesity expressed shock and concern about the *emotion of checking OGTT* (4.2). All participants underwent postnatal review to check the recovery; they did not know that an oral glucose tolerance test (OGTT) was required. Moreover, most women with GDM were unwilling to undergo OGTT due to their dislike of the sugar water, long blood drawing time, and pain. They believed that there was no need for further blood glucose management. Even when OGTT was abnormal, they thought that a healthy lifestyle was considered a long-term goal rather than now.

This study found that women with GDM and overweight/obesity lacked attention to postpartum blood glucose screening and *knowledge acquisition* for diabetes prevention (4.3). Participants reported that when diagnosed, obstetricians told them to pay attention to diet and exercise to control blood glucose levels and reduce the possible risks. But they did not believe that GDM is a serious problem. Meanwhile, no participants received specific information or counseling from healthcare professionals about postpartum self-health management after delivery. Thus, a disconnection was observed between disease prevention and health management.

## Discussion

In this study, we interviewed overweight/obese women with a history of GDM. We obtained information on their feelings, knowledge, experience, and behavior regarding diet, physical activity, family support needs, and future disease risk during puerperium. Lifestyle habits related to cultural background and family environment may adversely impact the recovery of β-islet cell function during postpartum. Overweight/obese women with GDM are at a higher risk or younger of developing T2DM in the long run, owing to the deterioration of β-islet cell function (Berggren et al., 2015). However, women with GDM are poorly informed about the risk of long-term metabolic diseases associated with being overweight/obese. Following delivery, participants at different education levels and professions had similar unhealthy eating and physical inactivity behaviors. Simultaneously, participants had a low awareness of postpartum disease prevention and healthcare due to the lack of information provided by health professionals and were more likely to neglect a healthy lifestyle.

Twelve of the 14 obese or overweight women who gained excessive gestational weight during pregnancy reported misconceptions about food, a lack of self-control, and excessive food intake high in fat and sugar. Studies in Sweden and Singapore found that women with a history of GDM had unhealthy diet habits after delivery (Persson et al., 2015; Teh et al., 2021). The participants ate more after delivery than during pregnancy because they believed rich and abundant foods were necessary to promote postpartum recovery, breast milk production, and reduce “confinement” illness. Furthermore, they believed that changes in healthy eating patterns, including frequent and little eating, moderate fruit intake, and fatty soups, should be delayed until the end of breastfeeding or longer (McIntyre et al., 2020). However, the puerperium period is critical for the recovery of islet cell function, and culturally related unhealthy eating habits may have irreversible negative effects (Neven et al., 2022). Therefore, research exploring postpartum health guidance of culturally oriented and diabetes prevention is particularly important for overweight/obese women with a history of GDM.

Furthermore, we found that overweight/obese women felt that exercise was necessary to control blood glucose levels only during pregnancy but not postpartum. Gunn et al. (2020) showed that women’s experiences of GDM during pregnancy influenced postpartum monitoring and prevention to some extent. Women with a history of GDM believe that postpartum blood glucose will always return to normal, and the cognitive misunderstanding may be due to incomplete educational information about GDM during pregnancy (He et al., 2021). Simultaneously, the need for physical activity during puerperium in overweight/obese women contradicts the traditional cultural “confinement” behavior. “Confinement” advises women to avoid physical activity and spend extended periods of time in bed. A study on the postpartum lifestyle of women with GDM in Singapore also found maternal postnatal physical inactivity and lack of time to manage healthy lifestyles (Teh et al., 2021). This inactivity lifestyle is not conducive to controlling/reducing the retained gestational excess weight gain.

Women with GDM have self-image stigma and shame due to their overweight/obese, accompanied by adverse emotional reactions (Nagpal et al., 2021). In contrast, some women think they are healthy and do not need to care about their weight. Damm et al. (2016) found that most women were unaware that recent GDM and postpartum weight retention increased their risk of T2DM or premature aging. The special population of overweight/obese women should continue to receive reliable information on weight management, disease prevention programs, psychological counseling, and professional activities by healthcare providers starting from pregnancy to postpartum. This professional guidance is a prerequisite for postpartum weight control and physical exercise for Chinese women with GDM (Hedeager Momsen et al., 2021).

Furthermore, the burden of disease prevention and management and the lack of social support make it difficult for overweight/obese women to adopt healthy lifestyles. Krompa et al. (2020) also reported that most women in France have postpartum depression and cannot care for their health due to a lack of parenting knowledge or partner assistance. In this study, we showed that Chinese women with GDM expressed a desire to crave emotional support from their partners and a collaborative relationship with their mother-in-law, which may help reduce negative emotions. Moreover, Bernstein et al. (2016) reported that neonatal care and breastfeeding was the most common barrier among respondents; thus, they did not get time for continuous postpartum monitoring of their health. The present study also found challenges in willingness and implementation to increase physical activity beyond a lack of time.

Researchers have observed that an unhealthy lifestyle during puerperium was common among overweight/obese women with a history of GDM (Hedeager Momsen et al., 2021; Neven et al., 2022). Of course, this lifestyle is influenced by cultural beliefs about “confinement” behavior and a lack of disease risk perception (Shang et al., 2021). Risk perception is influenced by many factors, such as personal experience, family support, and available professional information (Ferrer and Klein, 2015). It is a prerequisite for health-promoting behavioral change, which is crucial in positive health outcomes (Mukerji et al., 2016; Teh et al., 2021). Thus, systematic follow-up programs are ideal for preventing the progression of GDM to diabetes, but unfortunately, such planning is lacking in routine clinical settings in China.

Poor awareness of metabolic disease risks among the participants, particularly the risk of T2DM, was associated with limited or lack of lifestyle advice from health professionals (Timm et al., 2021). The discharge guidance recommends that overweight/obese women with a history of GDM be educated on incorporating healthy lifestyle practices during the puerperium or long-term postnatal period. Lifestyle practices should include diet, physical activity, weight management, and disease risk knowledge. Professional guidance can help high-risk women to control weight or promote loss, improve endocrine and metabolic status, and reduce the risk of developing T2DM at a younger age (Goveia et al., 2018).

This study demonstrates the need to enhance the education of overweight/obese women about healthy lifestyles and diabetes risk from prenatal to long-term follow-up. However, overweight/obese women with GDM currently face barriers to accessing continuing care from pregnancy to postpartum. Long-term health promotion and preventive care are essential to prevent chronic diseases, particularly type 2 diabetes (Reach, 2019). Therefore, the present study highlights the need to continue postpartum healthcare services, including improving the community service system currently facing challenges in China.

## Strength and limitations

The key strength of the present study includes understanding the lifestyle of women with overweight/obese women and a history of GDM during puerperium in China. The findings suggest the importance of postnatal primary care for overweight/obese women at high risk of type 2 diabetes who are unaware of the importance of lifestyle on blood glucose. Simultaneously, compliance with postpartum screening OGTT is low, and the education on blood glucose screening must be improved.

Also, the present study has certain limitations that influenced the findings. First, all participants were ethnically and geographically homogenous. The puerperal lifestyle is related to the cultural practice of “confinement” and may not reflect the experience of other ethnic minorities or regions. Further research on lifestyle similarities and differences among diverse populations is required from diverse cultures, races/ethnicities, geographic regions, etc. Second, social factors such as the respondents’ educational background and economic status may impact their experience. Thus, further studies are required to investigate stratified analyses of social lifestyle determinants and experiences in overweight/obese women during puerperium.

## Conclusion

The results obtained from this study provide information about dietary habits, weight perception, physical activity, and health knowledge of overweight/obese women with GDM during puerperium. According to this study, these women commonly had unhealthy lifestyles, misconceptions about food, a conflict between physical activity and confinement behavior, lack of social and family support, and low awareness of disease risk. Nevertheless, lifestyle-related blood glucose levels may be a key factor in preventing or delaying T2DM. Therefore, we emphasized the importance of healthcare providers providing continuous preventive care from pregnancy to postpartum and promoting long-term health in high-risk populations with a history of GDM associated with overweight/obesity.

## Data availability statement

The datasets presented in this article are not readily available due to the sensitivity of the questions asked, and the assurance to respondents that the original data would be kept confidential, and that the full transcript of the interview content would not be shared. Requests to access the datasets should be directed to tjwhhlb@126.com.

## Ethics statement

The studies involving human participants were reviewed and approved by Medical Ethics Committee of Tongji Hospital, Tongji Medical College of Huazhong University of Science and Technology. The patients/participants provided their written informed consent to participate in this study.

## Author contributions

JH, KH, CX, BW, TZ, and HW performed the study concept and design. JH and CX conducted the interviews. JH, KH, and TZ provided substantial contributions to the interpretation of data. HW performed the administrative, technical, or material support. All authors had full access to all data in the study and are responsible for the integrity of the data and the accuracy of the data analysis and involved in critical revision of the manuscript for important intellectual content.

## Funding

This work was supported by Wuhan Nursing Association in China (grant number, WHHL202201).

## Conflict of interest

The authors declare that the research was conducted in the absence of any commercial or financial relationships that could be construed as a potential conflict of interest.

## Publisher’s note

All claims expressed in this article are solely those of the authors and do not necessarily represent those of their affiliated organizations, or those of the publisher, the editors and the reviewers. Any product that may be evaluated in this article, or claim that may be made by its manufacturer, is not guaranteed or endorsed by the publisher.
